# Supplementary material for: A deep learning algorithm with good prediction efficacy for cancer-specific survival in osteosarcoma: A retrospective study
Source: PLoS One. 2023 Sep 28;18(9):e0286841. doi: 10.1371/journal.pone.0286841 (PMC10538762; doi:10.1371/journal.pone.0286841)
Supplement: Attachment — Submitted filename: A deep learning algorithm with good prediction efficacy for cancer-specific survival in osteosarcoma.pdf [file pone.0286841.s001.pdf]

A deep learning algorithm with good prediction efficacy for cancer-specific survival in osteosarcoma: a retrospective study.

Yang Liu, Lang Xie, Dingxue Wang , Kaide Xia

In this study the authors developed a high-performance prediction model for CSS rates in patients with OSC, using the DeepSurv algorithm. In addition, the developed model was deployed on a webpage to provide physicians and patients with an easy-to-use management prediction tool to facilitate personalized treatment.

The manuscript is well presented, the topics innovative and well described. The tables and graphs are clear and sufficient. Only a few aspects would need to be revised by the authors:

- It would be advisable to include the abbreviations in the abstract (lines 28-29).
- It is suggested that the authors include information on the epidemiology, staging and diagnosis, tumour location and mortality rate of OSC in the introduction. In this regard, the authors may also refer to the following work: de Nigris F, Rossiello R, Schiano C, Arra C, Williams-Ignarro S, Barbieri A, Lanza A, Balestrieri A, Giuliano MT, Ignarro LJ, Napoli C; Deletion of Yin Yang 1 Protein in Osteosarcoma Cells on Cell Invasion and CXCR4/Angiogenesis and Metastasis. *Cancer Res* 15 March 2008; 68 (6): 1797–1808. https://doi.org/10.1158/0008-5472.CAN-07-5582.
- It would be appropriate to clarify in the discussion the possible future developments of the project and how to proceed in order to solve the limitations of this study.
