# Supplementary material for: A deep learning algorithm with good prediction efficacy for cancer-specific survival in osteosarcoma: A retrospective study
Source: PLoS One. 2023 Sep 28;18(9):e0286841. doi: 10.1371/journal.pone.0286841 (PMC10538762; doi:10.1371/journal.pone.0286841)
Supplement: Attachment — Submitted filename: Response to reviewers.pdf [file pone.0286841.s002.pdf]

Reviewer #1

•It would be advisable to include the abbreviations in the abstract (lines 28-29).

Response: Dear Prof. Thanks for your valuable comments. We have added the abbreviations of the relevant terms in the abstract. I would appreciate your review of the revised manuscript. **(In red, the same below)**

•It is suggested that the authors include information on the epidemiology, staging and diagnosis, tumour location and mortality rate of OSC in the introduction. In this regard, the authors may also refer to the following work: de Nigris F, Rossiello R, Schiano C, Arra C, Williams-Ignarro S, Barbieri A, Lanza A, Balestrieri A, Giuliano MT, Ignarro LJ, Napoli C; Deletion of Yin Yang 1 Protein in Osteosarcoma Cells on Cell Invasion and CXCR4/Angiogenesis and Metastasis. Cancer Res 15 March 2008; 68 (6): 1797–1808. https://doi.org/10.1158/0008-5472.CAN-07-5582

Response: Thank you very much for the excellent reference you recommended, from which we have learned a lot of professional knowledge. The content related to OSC epidemiology is described in the text. We request you to review it again *(Line 42-44)*.

•It would be appropriate to clarify in the discussion the possible future developments of the project and how to proceed in order to solve the limitations of this study.

Response: We have discussed experimental designs that may address manuscript flaws in the future *(Line 233-234)*. Please review it and feel free to contact us if there are any inappropriate points. We look forward to more guidance from you.

Reviewer #2

The study is aimed to predict the cancer-specific survival rate in patients with OSC using deep learning algorithms and classical Cox proportional hazard models to provide data to support individualized treatment of patients with OSC. Data derived from patients diagnosed with OSC from 2004 to 2017 and obtained from the Surveillance, Epidemiology, and End Results database. The study sample was then divided randomly into a training cohort and a validation cohort in the proportion of 7:3. The DeepSurv algorithm and the Cox proportional hazard model were chosen to construct prognostic models for patients with OSC. A total of 3218 patients were randomized into training and validation groups (n = 2252 and 966, respectively). Both DeepSurv and Cox models had better efficacy in predicting cancer-specific survival (CSS) in OSC patients (C-index >0.74). In the validation of other metrics, DeepSurv did not have superiority over the Cox model in predicting survival in OSC patients.

I found the study of interest. The methodology is correct. Data are well presented and results support conclusions.

Response: Dear Professor, Thank you for your comments. If there is anything else in the manuscript that is not appropriate, please feel free to contact us. We look forward to more guidance from you. Thanks again.
